# Dentin Bond Integrity of Hydroxyapatite Containing Resin Adhesive Enhanced with Graphene Oxide Nano-Particles—An SEM, EDX, Micro-Raman, and Microtensile Bond Strength Study

**DOI:** 10.3390/polym12122978

**Published:** 2020-12-14

**Authors:** Yasser F. AlFawaz, Basil Almutairi, Hiba F Kattan, Muhammad S. Zafar, Imran Farooq, Mustafa Naseem, Fahim Vohra, Tariq Abduljabbar

**Affiliations:** 1 Department of Restorative Dental Sciences, College of Dentistry, King Saud University, 60169, Riyadh 11545, Saudi Arabia; yalfawaz@ksu.edu.sa (Y.F.A.); balmutiri@ksu.edu.sa (B.A.); 2 Preventive Dental Science Department, Princess Nourah bint Abdulrahman University, Riyadh 11545, Saudi Arabia; hfkattan@pnu.edu.sa; 3 Department of Restorative Dentistry, College of Dentistry, Taibah University, Al Madinah, Al Munawwarah 41311, Saudi Arabia; mzafar@taibahu.edu.sa; 4 Department of Dental Materials, Islamic International Dental College, Riphah International University, Islamabad 44000, Pakistan; 5 Faculty of Dentistry, University of Toronto, Toronto, ON M5S 2E8, Canada; Imran.farooq@mail.utoronto.ca; 6 Department of Community and Preventive Dental Sciences, College of Dentistry Dow International Dental College, Karachi 75500, Pakistan; Mustafa.naseem@duhs.edu.pk; 7 Department of Prosthetic Dental Science, College of Dentistry; Research Chair for Biological Research in Dental Health, College of Dentistry, King Saud University, Riyadh 11545, Saudi Arabia; fvohra@ksu.du.sa

**Keywords:** nano-hydroxyapatite, graphene oxide, dentin adhesive, microtensile bond strength, SEM-EDX, micro-Raman

## Abstract

The aim was to synthesize and characterize an adhesive incorporating HA and GO nanoparticles. Techniques including scanning electron microscopy (SEM) and energy dispersive X-ray spectroscopy (EDX), micro-tensile bond strength (μTBS), and micro-Raman spectroscopy were employed to investigate bond durability, presence of nanoparticles inside adhesive, and dentin interaction. Control experimental adhesive (CEA) was synthesized with 5 wt% HA. GO particles were fabricated and added to CEA at 0.5 wt% (HA-GO-0.5%) and 2 wt% GO (HA-GO-2%). Teeth were prepared to produce bonded specimens using the three adhesive bonding agents for assessment of μTBS, with and without thermocycling (TC). The adhesives were applied twice on the dentin with a micro-brush followed by air thinning and photo-polymerization. The HA and GO nanoparticles demonstrated uniform dispersion inside adhesive. Resin tags with varying depths were observed on SEM micrographs. The EDX mapping revealed the presence of carbon (C), calcium (Ca), and phosphorus (P) in the two GO adhesives. For both TC and NTC samples, HA-GO-2% had higher μTBS and durability, followed by HA-GO-0.5%. The representative micro-Raman spectra demonstrated D and G bands for nano-GO particles containing adhesives. HA-GO-2% group demonstrated uniform diffusion in adhesive, higher μTBS, adequate durability, and comparable resin tag development to controls.

## 1. Introduction

Dental resin composites are among the most utilized restorative materials in dentistry due to superior aesthetics and improved mechanical properties [1]. Dentin adhesives are employed to bond hydrophobic resin composites to hydrophilic dentin tissue, for dentin preservation and caries resistance [2]. Adhesion with dentin is more challenging for dental adhesives as dentin is less mineralized with high water content compared to enamel, thus warranting more technique sensitivity [3]. The quality of the adhesion with dentin depends on the ability of monomers to penetrate inter-collagen fiber spaces and stable resin tags to establish a hybrid layer [4]. One major reason for the composite restorative failure is loss of adhesive bond over a period of time [5]. This loss of bond leads to the formation of nano-gaps resulting in bond failure and development of secondary caries [6]. To overcome failure of adhesion over time, addition of inorganic fillers in adhesives is promoted to improve dentin interaction of adhesive resins and possibly minimize dentin-adhesive degradation [7]. Previous studies have shown that addition of fillers in adhesives can reduce water sorption and solubility [8], in addition to enhancing its mechanical properties [9]. Therefore, addition of inorganic nano-materials as fillers to develop the physical and mechanical properties of dentin adhesive’s is highly desirable.

Graphene is one material that has caught the attention of dental researchers in recent years. Graphene based materials possess high surface area and are chemically and thermally stable [10]. Among the graphene family nanomaterials (GFNs), graphene oxide (GO) is of particular interest and it can be synthesized by the oxidation of graphite [11]. In general, graphene is considered a hydrophobic material but GO, when compared with other GFNs, is considered hydrophilic due to the occurrence of oxygen in its functional groups [12]. This hydrophilicity could be considered an advantageous property as it helps GO to form steady colloid dispersion and evade aggregation, thus making it more cytocompatible [13]. An earlier study conducted by He et al., assessed the efficacy of GO nanosheets in inhibiting the growth of *P. gingivalis*, *F. nucleatum*, and *S. mutans.* It was reported that GO was able to inhibit the growth of these common dental pathogens effectively [14]. GO based materials are also used for scaffold formation and in a previous study by Nishida et al., it was reported that the insertion of GO based scaffolds in the tooth extraction sockets of dogs led to five times more rapid bone formation than collagen based scaffolds [15]. The coating of dental implants with graphene-based materials also decreases the adhesion of microorganisms, preventing implant failure [11]. GO as compared with graphite retains higher antimicrobial activity and has been added previously in dentin adhesive successfully by Lee et al. although they added a combination of GO and bioactive glass [16]. We also wanted to test the effect of addition of GO on the properties of adhesive but with another remineralizing agent called hydroxyapatite (HA).

HA is an inorganic, non-toxic bioactive material, commonly occurring as a main constituent of tooth structure and available in the form of nano particles. [17]. Considering the proven remineralization property of HA, its inclusion in many dental materials has increased [18]. As the dental adhesives come in direct contact with the tooth dentin, incorporation of HA nano particles in resin adhesives can possibly remineralize the caries affected dentin. In addition, interaction of HA within the hybrid dentin layer forming an organic bond, will contribute positively to its bond strength to tooth [19]. In an earlier study, Leitune et al. reported that inclusion of nano-HA particles in the experimental adhesive improved its bond strength to dentin [20].

The testing of mechanical properties of dental materials is essential in order to come up with an ideal material that can survive severely dynamic oral environment. Khosravani performed a study previously and inspected dental restorative materials after static and impact loadings [21]. This study can be used as a guide to develop new materials and perform their computational analysis. Another useful model was put-forward by Carreon and Funkenbusch [22] where they shed light on the role of machinable ceramics in restoration of teeth. This model also predicted, how the mechanical properties of a material will affect its abrasive grinding. These methods [21,22] are useful and should be kept in mind by researchers while synthesizing new dental materials.

In an earlier study, Bin-Shuwaish et al., demonstrated that addition of GO particles could improve mechanical properties of adhesive [23]. Mei et al., in a similar previous study revealed that addition of 1 wt% GO-silica particles could improve the compressive strength of experimental adhesives [24]. This encouraged us to see the effect of incorporation of two different concentrations (0.5 wt% and 2 wt%) of GO particles on various properties of adhesive. In line with these studies [23,24], we wanted to synthesize an adhesive with GO particles but in comparison, we also wanted to add HA particles in the adhesive first to utilize beneficial properties of these two nanomaterials (HA and GO).

An experimental dentin resin based adhesive incorporated with HA coated GO particles, could potentially enhance the mechanical bond strength durability along with remineralization potential of the resin dentin bond. Therefore, it is hypothesized that the incorporation of increasing content of GO particles will improve the bond strength, durability, and dentin interaction of experimental dentin adhesive having HA. Therefore, the aim of this study was to synthesize and characterize an adhesive incorporating HA and GO particles, using scanning electron microscopy (SEM) and energy dispersive X-ray spectroscopy (EDX). Also, to incorporate different contents of GO in experimental dentin adhesive (containing HA) and assess its micro-tensile bond strength (μTBS), bond durability, and dentin interaction.

## 2. Materials and Methods

### 2.1. Preparation of Experimental Adhesive

The experimental adhesive was synthesized utilizing the methods recommended earlier by Ye et al. [25]. Briefly, a mixture of monomers comprising of bisphenol A glycol dimethacrylate (BisGMA), triethylene glycol dimethacrylate (TEGDMA), 2-hydroxyethyl methacrylate (HEMA), and ethyl 4-dimethylamino benzoate and camphorquinone (Esstech Inc., Essington, PA, USA) were used. Our preparation contained a combination of w/w 50% BisGMA, 25% TEGDMA and 25% HEMA (60%) with ethanol acting as a solvent (30%-m/m). Also, we incorporated 0.5% (n/n) ethyl 4-dimethylamino benzoate and 0.5% camphorquinone photo-initiators in line with the monomer moles. Furthermore, 1.0% (n/n) diphenyliodonium hexafluorophosphate (DPIHP) was added to act as an electron initiator to the adhesive mix. This mixture was produced in a three-necked flask having a magnetic stirrer and condenser (SA300; Sansyo, Tokyo, Japan). To avoid photo-polymerization, the newly synthesized adhesive was preserved in an isolated dark chamber shielded with a foil.

### 2.2. Addition of HA to the Experimental Adhesive

The nano-HA particles (hydroxyapatitie—Sigma Aldrich, Cassopolis, MI, USA) were commercially acquired and silanized. To promote adhesion of nano-HA to the adhesive, 5% silane was added in the 95% acetone solvent, for silanization. The nano-HA particles were added in 5% concentration (m/m) to the experimental adhesive in order to obtain our control experimental adhesive (CEA) containing 5 wt% nano-HA particles. To promote homogenization of nano-HA particles in the experimental adhesive, dispersion of HA by means of sonication in a centrifuge was completed. The synthesized adhesives were kept isolated at 37 °C for 24 h to permit solvent evaporation. The adhesives were then kept at 4 °C and were used within 20 days of their formulation due to their narrow shelf life.

### 2.3. Synthesis of GO and Addition to the Experimental Adhesive

The GO was produced from natural graphite powder following the methods recommended earlier by Hummers and Offeman [26]. The step-by-step procedure for the GO’s formulation was also adopted from an earlier study of Deshmukh et al. [27]. Briefly, 3 gm of graphite powder and sodium nitrate (NaNO_3_) each were charged in a three-neck flask and mixed with 150 mL of sulphuric acid (H_2_SO_4_) with magnetic stirring. This newly formulated solution was cooled off at 5 °C for 5 h. This was followed by the addition of 9 gms of potassium permanganate (KMnO_4_) with continuous stirring. The oxidation of graphite was performed for 48 h at 40 °C and dilutions for the mix included adding of 150 mL distilled water and 30 mL of 30% hydrogen peroxide (H_2_O_2_). Lastly, the contents were washed, filtered, and centrifuged for de-acidification. The GO powder was made by vacuum drying for 60 °C for 13 h. The silanization of GO was executed to augment its interface and adhesion to resin matrix, by adding 5% silane to the 95% acetone solvent [28].

The GO powder was mixed in 2 mL ethanol in microvial and sonicated for 10 min at 37 °C in an ultrasonicator (VWR USC-TH sonicator bath, Tokyo, Japan). GO powder was added to the ethanol solvent of the formulated CEA at 0.5% and 2.0% to produce two additional experimental adhesives; HA-GO-0.5% and HA-GO-2%, respectively. In order to achieve a homogenous mixture, the nano-GO particles were initially mixed in resin and sonicated in an ultrasonic bath (VWR USC-TH sonicator bath, Tokyo, Japan) for 10 min. Post-sonication, the mixture was homogenized in an ultrasonic homogenizer (Q500 Sonica) at pulse on/off for 60 s at room temperature. To warrant that GO was evenly disseminated after storing, the mix was re-homogenized in the ultrasonic homogenizer on every single usage. The weight of the nanoparticles was calculated in milligrams and the volume of resin in milliliters. With the intention of calculating 0.5 w/v % adhesive (HA-GO-0.5%) and 2.0 w/v % adhesive (HA-GO-2%), the following formula were used.

Weight/volume % = weight of solute/volume of solution × 100

The components of the new adhesives produced are shown in Table 1. The new adhesives produced were stored at 4 °C and were used within 2 weeks of their formulation.

### 2.4. Scanning Electron Microscopy (SEM) Analysis

The scanning electron microscopy (SEM) was utilized to assess the presence of nano-GO particles and their interaction inside the resin adhesive. Five samples each for HA-GO-0.5% and HA-GO-2% experimental adhesives were formulated post-photo-polymerization with a dental curing light source (Curing Light, Eliphar S10; 3M ESPE, St. Paul, MN, USA) with 600 mW.cm^−2^ output for 20 s at a distance of 10 mm. Prior to performing SEM, specimens mounted on aluminum stubs and sputter coated with gold layer for 120 s (Baltec SCD sputter, Scotia, NY, USA). The analysis was completed using SEM (JEOL, JSM-6513, SEM, Tokyo, Japan) at an accelerating voltage of 30 kV. The SEM micrographs were taken at multiple magnifications for convenience.

### 2.5. Preparation of Teeth Specimens

One hundred and two extracted non-carious maxillary premolars (*N* = 102) which were extracted for orthodontic reasons, were collected from Oral Surgery clinics of the institute. The teeth were cleaned employing an ultrasonic scaler (Superior Instruments Co, New York, NY, USA). The teeth were disinfected by immersing them in chloramine trihydrate solution (Merck, Germany) and then stored in distilled water at 4 °C. These teeth were then implanted vertically with the help of acrylic resin (Opti-Cryl, South Carolina, Columbia) in segments of polyvinyl tubes (4 mm diameter) at the cementoenamel junction (CEJ).

The occlusal surface of all the teeth were removed with a diamond saw (Buehler Isomet 2000 Precision saw, IL, USA) to expose dentin tissue 1 mm underneath the dentinoenamel junction (DEJ). An area of sound dentin (~5 mm) was recognized in the middle part of the occlusal surface and treated with 36% phosphoric acid (DeTrey conditioner, Dentsply, PA, USA) for 10 s followed by washing with distilled water and drying with cotton pellets. These teeth were equally and randomly allocated to three adhesive groups and 34 teeth each (*n* = 34) were then treated with formulated adhesives; Gp-1: teeth treated with CEA containing 5% HA, Gp-2: teeth treated with HA-GO-0.5% containing 5% HA (HA-GO-0.5%) and 0.5% GO particles, and Gp-3: teeth treated with HA-GO-2% containing 5% HA and 2% GO particles (HA-GO-2%). The adhesives were first dispensed on a disposable mixing pad and applied on the treated dentin with a micro-brush for 15 s trailed by air thinning for 5 s. The second application was performed in an identical manner to all the specimens. This was then followed by photo-polymerization of samples for 20 s at 10 mm distance using a curing device (Curing Light Eliphar S10; 3M ESPE, St. Paul, MN, USA).

The adhesive smeared samples were then enclosed with resin composite (Filtek Supreme; 3M ESPE, St. Paul, MN, USA) increments of 4 mm height in 2 mm augmentations using an acrylic jig, plastic instrument, and condenser. The excess material was detached and the interface was light cured from all sides for 20 s each. These bonded tooth samples were kept in distilled water at 37 °C for 1 week.

### 2.6. Microtensile Bond Test (μTBS) and Failure Mode Analysis

For microtensile bond testing (μTBS), sixty samples were used (twenty from each group). Out of 20 samples in each group, 10 bonded specimens in all adhesive groups were thermocycled (TC) in distilled water baths at 5 °C and 55 °C for 30 s with 5 s dwelling time (THE-1100, SD Mechatronik GmbH, Germany) and 10,000 cycles were used. The remaining 10 samples remained non-thermocycled (NTC) and were kept in distilled water for 24 h prior to sectioning. The bonded samples in each adhesive group were partitioned in order to construct 1 × 1 mm beams of composite–adhesive-dentin using a diamond slow speed saw (Buehler Isomet 2000 Precision saw, Rapid, IL, USA). Every group involved 20 teeth and each tooth formed six beams. In every adhesive group, at least five bonded beams were assessed for μTBS analysis. The beams were attached to the jaws of micro tensile tester (Bisco Inc., VA, USA) with cyanoacrylate (Zapit, Dental ventures Inc., Corona, CA, USA) and loaded in tension at 0.5 mm/min crosshead speed up to fracture. The failure modes were assessed in each group, and were classified as adhesive, cohesive, and mixed types by means of a digital microscope (Hirox KH 7700, Tokyo, Japan). The observed μTBS values in every group were matched using ANOVA and multiple comparisons test.

### 2.7. Micro-Raman Spectroscopy Analysis

The micro-Raman spectroscopy analysis of the dentin-adhesive bonded specimens was performed utilizing 21 bonded teeth (seven from each group) which were segmented to form beams for interfacial valuation of the samples in the three groups. A micro-Raman spectrophotometer (ProRaman-L Analyzer; TSI, Shoreview, MN, USA) with its software (Raman reader) was used to obtain Raman spectra(s). The alterations for dark counts were commenced with sample positioning modifications to attain satisfactory signals. The laser beam was fixated using a 0.9 objective lens and 600 mW power on the adhesive interface. The specimens were partitioned into segments of 1 mm and a 60 s scan was executed three times for the designated sample area. The particulars of the spectrum were gained at a laser beam wavelength of 785 nm between 800 cm^−1^ to 1800 cm^−1^ with noise filtration.

### 2.8. SEM and Energy Dispersive X-ray (EDX) Spectroscopy

Twenty-one bonded teeth among the three study groups (seven teeth from each group) were sectioned (as explained previously) to produce bonded beams (1 × 1 mm) for SEM and energy dispersive X-ray (EDX) spectroscopy. These beams were wet-polished (Beuhler Polisher, Lake Bluff, IL, USA), washed and placed in ultrasonic bath containing distilled water for 5 min (Bandelin Digital-Sigma-Aldrich Darmstadt, Germany). This step was followed by conditioning with 36% phosphoric acid (DeTrey conditioner, Dentsply, PA, USA) trailed by washing and dipping in 5.25% sodium hypochlorite (NaOCl) solution for 15 min and washing with distilled water again. The samples were dehydrated by placing them in ethanol solution (80%, 90%, and 100%). The samples mounting and gold coating was completed as explained earlier. The observations of the interface for the samples in the three groups were achieved using SEM (JEOL, JSM-6513, SEM, Tokyo, Japan) functioning at 30 kV voltage at photomicrographs were obtained at multiple magnifications and EDX.

## 3. Results

### 3.1. SEM/EDX Analysis

**Figure 1:**
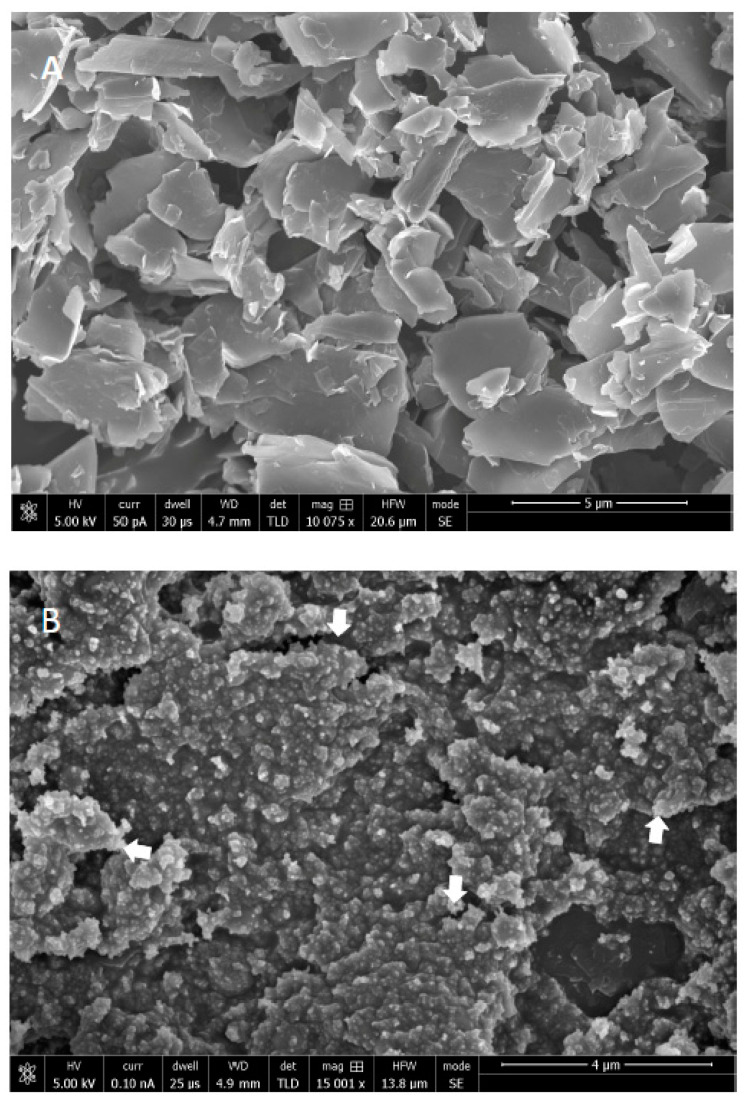

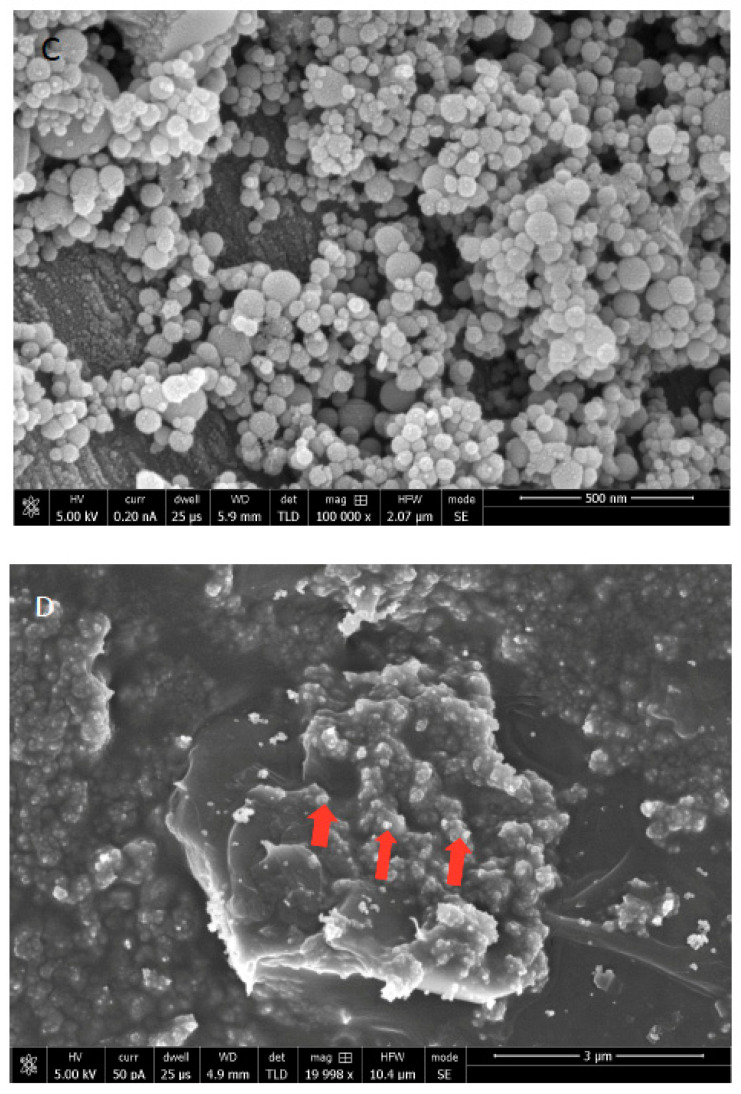
SEM images of (**A**) synthesized graphene oxide (GO) nanoparticles. Graphene oxide showing variable size flakes with 1–2 nm thickness. (**B**) Hydroxyapatite (HA) nanoparticles with <100 nm in size. These nanoparticles on SEM showing uniform round agglomerated structures. (**C**) Low magnification of composite structure of GO-HA, where HA nanoparticles were coated over reduced GO (white arrowhead). (**D**) High magnification of a single GO nanoparticle showing uniform distribution of HA (red arrowheads) over its surface.

The SEM analysis of synthesized -GO particles demonstrated 1–2 nm thick flakes (Figure 1a). The nano-HA particles demonstrated <100 nm agglomerated round structures deposited on GO flakes in SEM micrographs (Figure 1b). The mixture of HA with GO revealed coating of HA covering GO particles (Figure 1c). In terms of dispersion, uniform distribution of GO nanoparticles throughout the adhesive was observed (Figure 1d).

**Figure 2:**
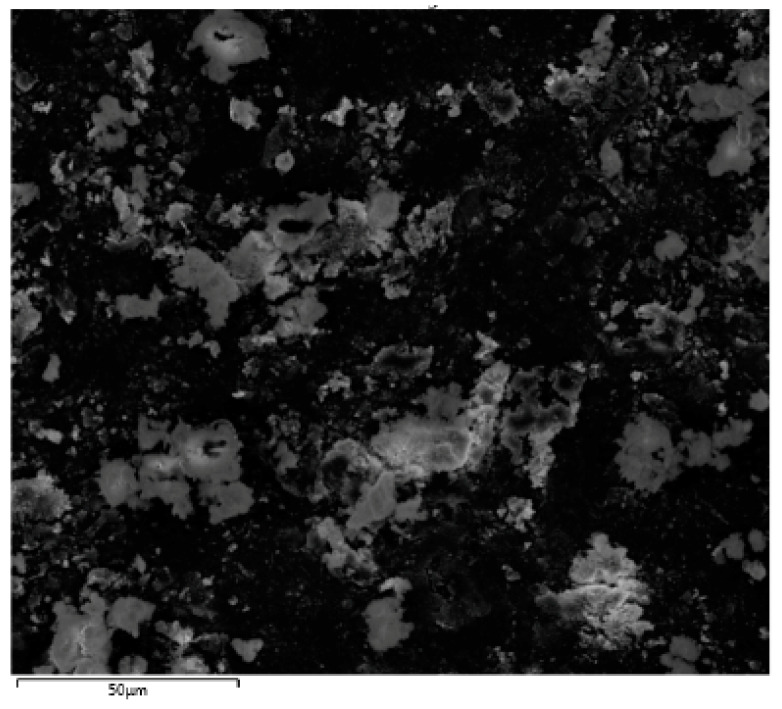

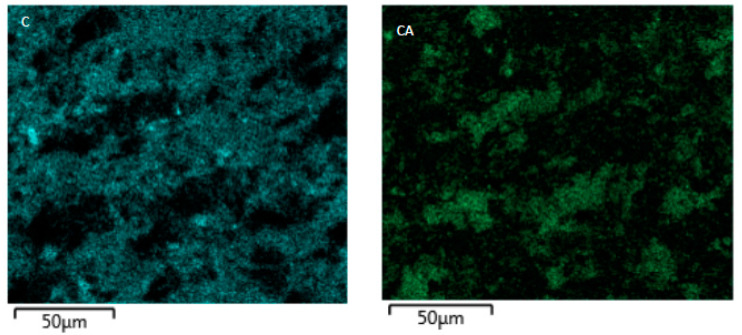

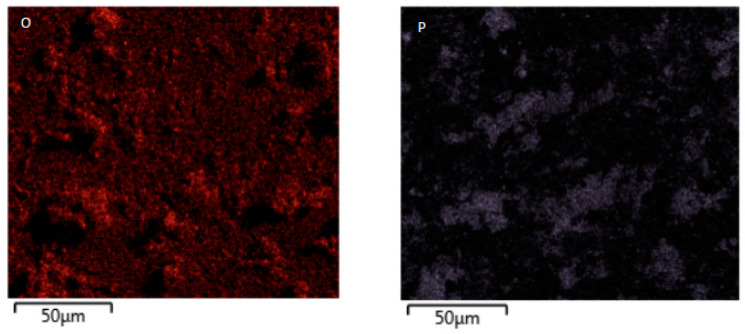
Representative electron image for EDS analysis of HA-GO-2.0% composite showing wide distribution of three important elements including carbon (C) as a representation of graphene, calcium (Ca) and phosphorus (P) evidencing the inclusion of HAP.

**Figure 3:**
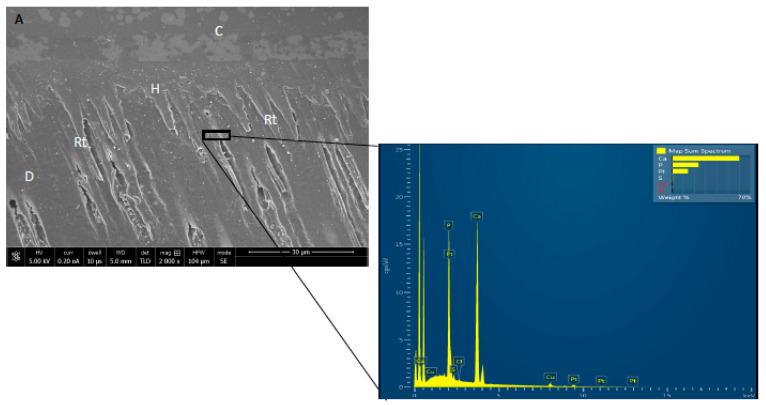

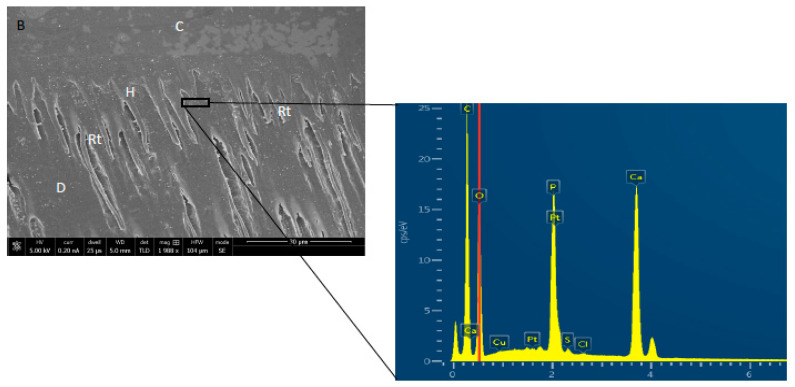
Resin-dentin bonded interface for (**A**) HA-GO-2.0% and (**B**) control showing excellent bonding between composite and dentin structure. EDX spectrum for HA-GO-2.0% specimen showing highest percentage of carbon (63.3%) followed by Calcium (10.8%) within the hybrid layer, reflecting the existence of graphene and HA nanoparticles. The control specimen containing highest percentage of calcium (70%) and phosphorus (30%) within the resin matrix. C = composite; H = hybrid layer; Rt = resin tags; D = dentin

The EDX investigation of HA and GO particle incorporated adhesives (HA-GO 2%) demonstrated the presence of carbon (C) in addition to calcium (CA) and phosphorus (P) (Figure 2). The presence of Ca and P indicates presence of HA, whereas C, warrants the presence of graphene. EDX mapping analysis of resin bonded interfaces demonstrated different results for the three groups. The highest percentage of C (63.3%) was seen in HA-GO-2% and in the same group, 10.8% Ca was also detected (Figure 3a). The highest percentage of Ca (70%) and P (30%) was seen in the CEA group and no C was noticed within the resin matrix (Figure 3b). Numerous resin tags at varying depths with a thick hybrid layer in dentin were also observed on SEM micrographs (Figure 3a,b).

### 3.2. Micro-Raman Spectroscopy Analysis

**Figure 4:**
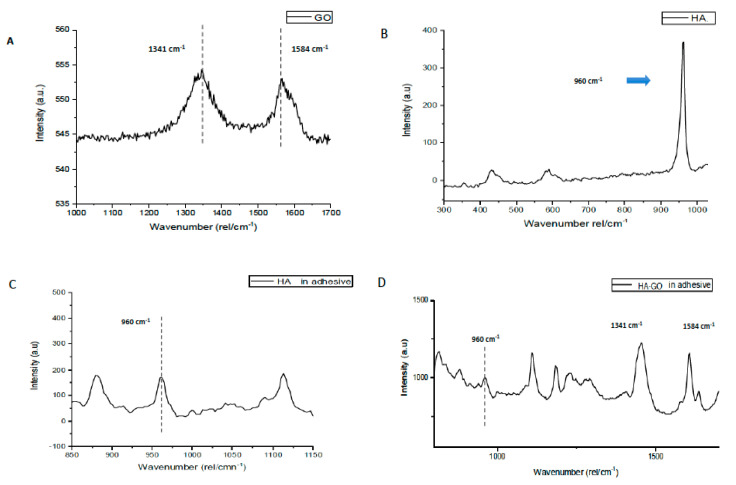
Raman spectra of (**A**) synthesized GO nanoparticles showing characteristic D and G bands which are two prominent peaks at 1341 and 1584 cm^−1^. Usually, the G mode is assigned to the vibration of sp2-hybridized carbon, while the D mode is related to the sp3-hybridized carbon and deficiencies relevant with grain boundaries and vacancies in GO. (**B**) Raman spectra of the hydroxyapatite nano particles showing chemical groups of phosphate (υ1 PO_4_ ~960 cm^−1^). (**C**) HAP within dentin adhesive showed weaker phosphate bonds intensities at 960 cm^−1^. (**D**) wiTH GO addition in dentin adhesive with HAP, the characteristic peaks could be seen for both nanoparticles at their respective wavenumbers.

The representative Raman spectra of GO particles, HA particles, HA in experimental adhesive (CEA), and HA-GO mixed in adhesive is shown in Figure 4A–D respectively. In Figure 4A, two vibrational bands are seen; the first band (D) was observed at 1341 cm^−1^ and the second band (G) was observed at 1584 cm^−1^. Typically, the D mode is linked to the sp3-hybridized carbon and insufficiencies related with grain boundaries and vacancies in GO, whereas, the G mode is allocated to the vibration of sp2-hybridized carbon. In Figure 4B, characteristic phosphorus-oxygen (P-O) peak at 960 cm^−1^ was observed for HA particles. Raman spectra for CEA are presented in Figure 4, showing distinctive P-O band at 960 cm^−1,^ however at a weaker intensity. For adhesive containing 5% HA and 2% GO, peaks within the Raman spectra (Figure 4D) were present at 960 cm^−1^ for P-O band representing HA; D and G bands were at 1341 cm^−1^ and 1584 cm^−1^ respectively (GO).

### 3.3. μTBS and Failure Mode Analysis Results

**Figure 5:**
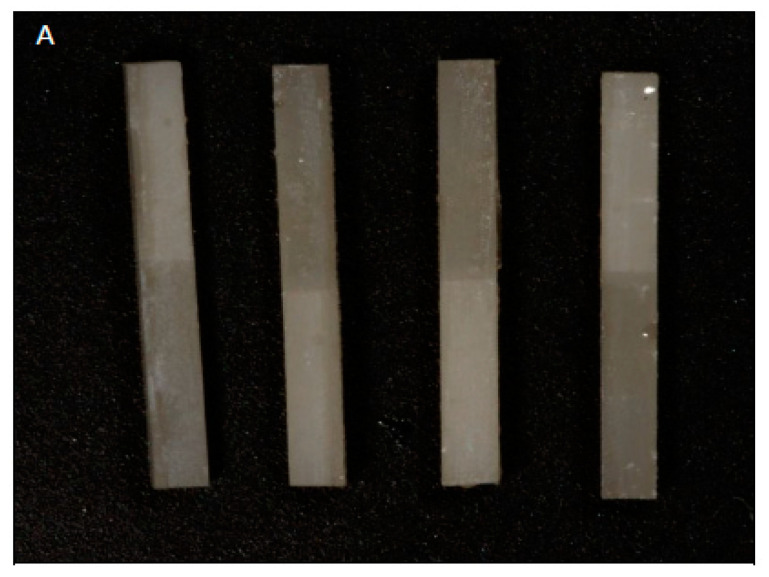

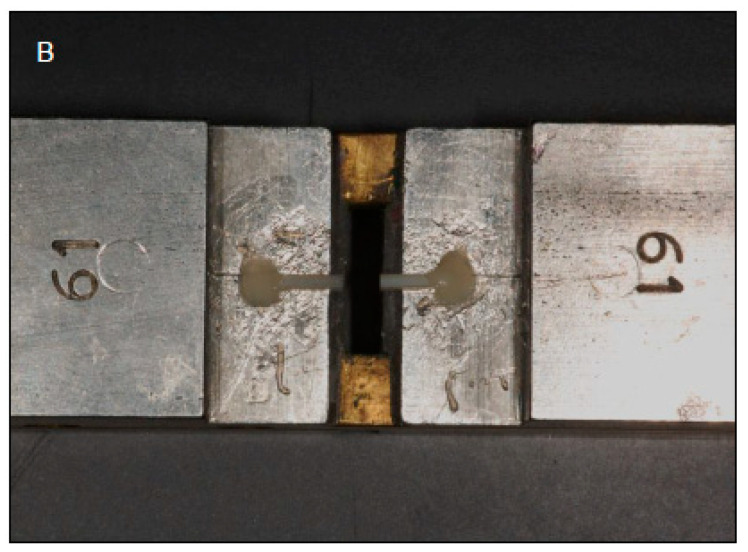
(**A**) Dentin bonded specimen sticks using experimental adhesives with 1 × 1 mm dimensions. (**B**) Debonded specimens after microtensile testing.

The μTBS (MPa) (Mean ± SD) observed for the samples (both TC and NTC) among three groups in this study along with their respective failure modes are shown in Table 2 (Figure 5A,B). It can be observed that for the NTC samples, the HA-GO-2% group had the greatest mean μTBS values (30.17 ± 3.63) followed by HA-GO-0.5% (29.74 ± 3.81) and CEA (25.21 ± 3.60) groups. For the TC samples, a similar pattern of μTBS was observed with NTC samples and HA-GO-2% showed higher bond strength (26.18 ± 3.11). On intergroup comparison for NTC samples, the differences observed for CEA (25.21 ± 3.60) compared with HA-GO-0.5% (29.74 ± 3.81); and CEA (25.21 ± 3.60) compared with HA-GO-2% (30.17 ± 3.63) were statistically significant (*p* < 0.01) respectively. For intergroup comparison of TC samples, the differences observed between CEA (21.77 ± 3.54) and HA-GO-0.5% (24.10 ± 3.37), and CEA (21.77 ± 3.54) matched with HA-GO-2% (26.18 ± 3.11) were statistically significant (*p* < 0.01) respectively. On intragroup comparison for TC and NTC samples, differences observed between CEA group compared with HA-GO-0.5% and those detected when CEA group was matched with HA-GO-2% group were all found to be statistically significant (*p* < 0.01) (Table 2). Overall, NTC samples revealed higher μTBS mean values compared to TC samples among all groups (*p* < 0.05). There was no clear distribution pattern observed for the failure modes observed among the three groups; however, the majority of the failures observed were of adhesive nature. Adhesive failure depicts a failure in adhesion with fractures which are not observed in dentin or the resin [29]. In our study, though adhesive type failures were most common, among them the smallest percentage of adhesive type failures were observed in NTC-HA-GO-0.5% (60%) and NTC-HA-GO-2% (60%) (Table 2).

## 4. Discussion

The present study investigated the influence of addition of nano-HA and different concentrations of nano-GO particles (0.5 wt% and 2 wt%) in an experimental resin adhesive on its bond integrity, durability and dentin interaction. Based on the results, HA-GO-2% group showed higher μTBS than HA-GO-0.5% group, which demonstrated higher μTBS than the controls (CEA). Therefore, the hypothesis that addition of GO nanoparticles in adhesive resin would improve its bond strength was accepted.

Dental adhesives play a vital role in the clinical success of restorations, as a weak restoration-adhesive bond is linked with microleakage, dentin hypersensitivity (DH), and secondary caries development [30]. The use of nanoparticles in general, reinforces various mechanical and physical properties of dental composites [31]. The nano-HA particles are biocompatible, act as a source of remineralizing ions like Ca and P, and retain strong capability to bind with the tooth tissues [32,33]. In the present study, we incorporated nano-HA particles in the experimental adhesive in order to yield all the benefits associated with the use of HA in the adhesives. It has been suggested earlier that the use of nano-HA particles improves the surface area for adhesion and strengthens the mechanical properties of adhesive [34]. In line with this recommendation, nano-HA particles (<100 nm in size) were incorporated in the present study. In addition, nano-GO particles in different concentrations (0.5% and 2.0%) were added to HA containing adhesive to further enhance adhesive bonding properties of the experimental adhesives.

The synthesized nano-GO particles were flake shaped, as observed via SEM in the study (Figure 1A). The reduction process of graphite to yield GO nanoparticles produces flake shaped particles or sheets with sharp edges due to the presence of oxygen groups in the oxide sheets, as reported previously by Soares et al. [35]. In order to utilize maximum benefit of the addition of foreign filler particles in the adhesive, it is necessary that it is uniformly dispersed. An incomplete or partial diffusion of filler nanoparticles could lead towards the formation of gaps or cracks, reducing ideal performance of the adhesive [36]. In the present study, SEM analysis revealed uniform dispersion of nano-HA particles (Figure 1B), and nano-GO coated with HA particles in the adhesive (Figure 1C,D).

The EDX analysis of adhesives in our study confirmed the occurrence of vital remineralizing elements like Ca and P in CEA and Ca, P, and C for HA-GO-0.5% group. These essential elements were again observed for the HA-GO-2% group (Figure 2). The presence of Ca and P indicates the presence of HA whereas C, warrants the presence of graphene. The Ca and P are crucial components of human hard tissues and are also essential for remineralization of dental tissues [37]. Dental plaque provides a niche where microorganisms can reside and cause dental caries and pulpal inflammation. Cantore et al., previously reported that where chlorhexidine is considered a standard antimicrobial mouthwash, an alcohol-free mouth wash has similar antimicrobial activity and can also prevent plaque formation [38]. Any ingredient of an oral product that makes it antibacterial and that can decrease microbial load, reduces the chances of dental caries and pulpal inflammation [38]. Presence of C in graphene based materials makes it antibacterial and its use in dental adhesive inhibits the adhesion of important dental pathogens including *Streptococcus mutans* [1]. In our study, the high percentage of C (63.3%) was observed in HA-GO-2% whereas, the highest percentages of Ca and P were detected in CEA group. Apart from ingredients that can make oral products antimicrobial, the role of stem cells in preventing inflammation of oro-dental tissues is also important and has attracted a lot of attraction recently. Spagnuolo et al. previously reported that stem cells can regenerate damaged tissues and can modulate inflammatory and immune responses [39]. Among stem cells from the oral cavity, dental pulp stem cells have shown promising regenerative properties [38]. Ballini et al. also reported that although there are barriers in the use of stem cells, they have still shown promising healing properties in various clinical environments [40]. Diniz et al. demonstrated that free radicals from dental adhesives could affect the survival properties of stem cells and laser phototherapy is actually useful in supporting stem cells existence [41]. Therefore, although this area was not investigated in our study, researchers should also look into the effect of dental adhesives on the stem cells in the future. Concerning resin tags, varying depths of these tags were also observed on SEM micrographs (Figure 3a,b). However, resin tag penetration in the tubules does not inevitably effect the bond strength and intactness of the adhesive, as reported by Anchieta et al. earlier [42].

Micro-Raman spectroscopy is a vibrational spectroscopic diagnostic system which is utilized to study polarization of molecules [43]. We engaged micro-Raman spectroscopy to study GO and HA nanoparticles as this technique involves scattering instead of absorption, therefore specimen of variable thicknesses can be observed without any destruction [44]. In addition, we also observed GO and HA nanoparticles after their inclusion in the adhesive via micro-Raman spectroscopy. For the nano-GO particles, two bands were observed where the first band (D) was seen at 1341 cm^−1^ and the second band (G) was observed at 1584 cm^−1^ (Figure 4A). Due to the energy shift caused by laser excitation, observing two peaks on Raman spectroscopy is a common observation for graphite based materials [45], as seen in our study. It is a common finding to observe the characteristic P-O band at 960 cm^−1^ for HA samples in Raman spectroscopy [46]. The typical P-O band was also observed for nano-HA particles at 960 cm^−1^ in our study (Figure 4B). When nano-HA particles were mixed in the adhesive, distinctive P-O band was still observed at 960 cm^−1^ but its intensity was weak (Figure 4C). The plausible reason of this particular finding could be that there is a transitional change in the intensity of the Raman bands when the minerals are substituted with resin, as reported formerly by Van Meerbeek et al. [47]. When the HA and GO particles were mixed together in adhesive, representative P-O band at 960 cm^−1^ indicating HA’s presence and D and G bands at 1341 cm^−1^ and 1584 cm^−1^ respectively representing GO’s presence were also seen.

We utilized μTBS to evaluate the bond strength of different experimental adhesives in the present study. The μTBS gives a better and precise estimation of bond strength as compared with other conventional techniques [48]. In an earlier study, Wagner et al., demonstrated that the adhesive bond strength could decrease if filler wt% of >10% is used, due to increase in viscosity of the material [49]. Keeping this in mind, we used 5 wt% of nano-HA filler in our adhesives. The results demonstrated higher μTBS values for HA-GO-2% as compared with the other groups. In an earlier study, Khan et al., also reported that inclusion of GO sheets improves the bond strength of commercial primers [50] and our results are in conformity with their study. In order to mimic the oral cavity’s dynamic conditions, bonded dentin-adhesive beams were TC to depict bond durability with temperature changes encountered in an in vivo environment. Earlier, Helvatjoglu-Antoniades et al. demonstrated that TC could decrease the bond strength of adhesives [51]. Their findings are in conformity with the present study, as all adhesive specimens demonstrated decreased μTBS due to TC [51].

Although promising results are obtained from this study where nano-GO particles were added in adhesive to positively reinforce properties of the adhesive, future studies exploring the influence of incorporating different concentrations of GO and their impact on mechanical and biological properties are suggested.

## 5. Conclusions

Within the limitations of the present study, it can be concluded that addition of nano-GO particles positively influenced mechanical properties of the experimental adhesive. HA-GO-2% group demonstrated uniform diffusion in experimental adhesive, good dentin interaction with hybrid layer formation, higher μTBS, and maximum bond durability among the tested adhesives. Therefore, inclusion of 2 wt% GO in HA containing adhesive could improve adhesives mechanical properties as it proved to be a promising filler in our study and could have practical applications in clinical dentistry. The exploration of different concentrations of GO for addition in HA adhesive could be considered as an advantage in our study. However, we explored only two GO concentrations, and this could be a potential disadvantage. While we probed only two concentrations of GO for addition in HA adhesive, our study can provide a tentative foundation for further analysis of other GO concentrations. Future work is warranted based on the results of our study to analyze the effect of addition of other GO concentrations on HA adhesive’s properties.

## Figures and Tables

**Table 1:** Components of the two new adhesives with GO nanoparticles produced for this study

Categories	CEA Resin Adhesive (5% HA)	Nanoparticles (mg)
HA-GO-0.5%	2 mL	10 mg
HA-GO-2%	2 mL	40 mg

CEA Resin adhesive composition- wt% (24% HEMA, 24% TEGDMA, 50% bis-GMA, 1% DPIHP, 0.5% EDB & 0.5% camphorquinone).

**Table 2:** Mean and SD of microtensile bond strength and failure modes among the tested study groups

	μTBS (MPa) (Mean ± SD)			Failure Mode Analysis (%)		
Group (*n* = 10)	NTC	TC	*p* Value *	Adhesive	Cohesive	Mixed
CEA	25.21 ± 3.60 ^aA^	-	<0.01	80	10	10
	-	21.77 ± 3.54 ^aB^		80	10	10
HA-GO (0.5%)	29.74 ± 3.81 ^bA^			60	20	20
	-	24.10 ± 3.37 ^bB^		80	10	10
HA-GO (2.0%)	30.17 ± 3.63 ^bA^			60	30	10
	-	26.18 ± 3.11 ^bB^		100	0	0

TC: thermocycling, NTC: no thermocycling, GO: graphene oxide, CEA: control experimental adhesive, * HA: hydroxyapatite. ANOVA. Dissimilar small alphabets in same column indicate statistical significance. Dissimilar capital alphabets in row (same group) indicate statistical significance.

## References

[1] Bregnocchi A., Zanni E., Uccelletti D., Marra F., Cavallini D., De Angelis F., De Bellis G., Bossu M., Ierardo G., Polimeni A. (2017). Graphene-based dental adhesive with anti-biofilm activity. J. Nanobiotechnol..

[2] De Almeida Neves A., Coutinho E., Cardoso M.V., Lambrechts P., Van Meerbeek B. (2011). Current concepts and techniques for caries excavation and adhesion to residual dentin. J. Adhes. Dent..

[3] Al-Hamdan R.S., Almutairi B., Kattan H.F., Alresayes S., Abduljabbar T., Vohra F. (2020). Assessment of Hydroxyapatite Nanospheres Incorporated Dentin Adhesive. A SEM/EDX, Micro-Raman, Microtensile and Micro-Indentation Study. Coatings.

[4] Huang B., Siqueira W.L., Cvitkovitch D.G., Finer Y. (2018). Esterase from a cariogenic bacterium hydrolyzes dental resins. Acta Biomater..

[5] Breschi L., Maravic T., Cunha S.R., Comba A., Cadenaro M., Tjaderhane L., Pashley D.H., Tay F.R., Mazzoni A. (2018). Dentin bonding systems: From dentin collagen structure to bond preservation and clinical applications. Dent. Mater..

[6] Ferracane J.L. (2017). Models of Caries Formation around Dental Composite Restorations. J. Dent. Res..

[7] Bin-Shuwaish M.S., Maawadh A.M., Alhamdan R.S., Alresayes S., Almohareb T., Almutairi B., Vohra F., Abduljabbar T. (2020). Influence of graphene oxide filler content on the dentin bond integrity, degree of conversion and bond strength of experimental adhesive. A SEM, micro-raman, FTIR and microtensile study. Mater. Res. Express.

[8] Kalachandra S. (1989). Influence of fillers on the water sorption of composites. Dent. Mater..

[9] Alshahrani A., Bin-Shuwaish M.S., Al-Hamdan R.S., Almohareb T., Maawadh A.M., Al Deeb M., Alhenaki A.M., Abduljabbar T.F. (2020). Graphene oxide nano-filler based experimental dentine adhesive. A SEM/EDX, Micro-Raman and microtensile bond strength analysis. J. Appl. Biomater. Fundam. Mater..

[10] Baig M.S., Fleming G.J. (2015). Conventional glass-ionomer materials: A review of the developments in glass powder, polyacid liquid and the strategies of reinforcement. J. Dent..

[11] Ge Z., Yang L., Xiao F., Wu Y., Yu T., Chen J., Lin J., Zhang Y. (2018). Graphene Family Nanomaterials: Properties and Potential Applications in Dentistry. Int. J. Biomater..

[12] Wei N., Lv C., Xu Z. (2014). Wetting of graphene oxide: A molecular dynamics study. Langmuir.

[13] Seabra A.B., Paula A.J., de Lima R., Alves O.L., Duran N. (2014). Nanotoxicity of graphene and graphene oxide. Chem. Res. Toxicol..

[14] He J., Zhu X., Qi Z., Wang C., Mao X., Zhu C., He Z., Li M., Tang Z. (2015). Killing dental pathogens using antibacterial graphene oxide. ACS Appl. Mater. Interfaces.

[15] Nishida E., Miyaji H., Kato A., Takita H., Iwanaga T., Momose T., Ogawa K., Murakami S., Sugaya T., Kawanami M. (2016). Graphene oxide scaffold accelerates cellular proliferative response and alveolar bone healing of tooth extraction socket. Int. J. Nanomed..

[16] Lee S.-M., Yoo K.-H., Yoon S.-Y., Kim I.-R., Park B.-S., Son W.-S., Ko C.-C., Son S., Kim Y.-I. (2018). Enamel Anti-Demineralization Effect of Orthodontic Adhesive Containing Bioactive Glass and Graphene Oxide: An In-Vitro Study. Materials.

[17] Farooq I., Moheet I.A., AlShwaimi E. (2015). In vitro dentin tubule occlusion and remineralization competence of various toothpastes. Arch. Oral Biol..

[18] Philip N. (2019). State of the Art Enamel Remineralization Systems: The Next Frontier in Caries Management. Caries Res..

[19] Kavrik F., Kucukyilmaz E. (2019). The effect of different ratios of nano-sized hydroxyapatite fillers on the micro-tensile bond strength of an adhesive resin. Microsc. Res. Tech..

[20] Leitune V.C., Collares F.M., Trommer R.M., Andrioli D.G., Bergmann C.P., Samuel S.M. (2013). The addition of nanostructured hydroxyapatite to an experimental adhesive resin. J. Dent..

[21] Khosravani M.R. (2019). Mechanical behavior of restorative dental composites under various loading conditions. J. Mech. Behav. Biomed. Mat..

[22] Carreon A.H., Funkenbusch P.D. (2019). Single-grain approach to material specific dental grinding-force equations. J. Manuf. Process..

[23] Qian W., Hu X., He W., Zhan R., Liu M., Zhou D., Huang Y., Hu X., Wang Z., Fei G. (2018). Polydimethylsiloxane incorporated with reduced graphene oxide (rGO) sheets for wound dressing application: Preparation and characterization. Colloids Surf. B..

[24] Mei L., Wei H., Wenjing C., Xiaokun H. (2017). Graphene Oxide-Silica Composite Fillers into the Experimental Dental Adhesives for Potential Therapy. Med. Res..

[25] Ye Q., Spencer P., Wang Y., Misra A. (2007). Relationship of solvent to the photopolymerization process, properties, and structure in model dentin adhesives. J. Biomed. Mater. Res. A.

[26] Hummers W.S., Offeman R.E. (1958). Preparation of graphatic oxide. J. Am. Chem. Soc..

[27] Deshmukh K., Pasha S.K., Deshmukh R.R., Bhagat P.R. (2015). Highly dispersible Graphene oxide reinforced polypyrrole/polyvinyl alcohol blend nanocomposites with high dielectric constant and low dielectric loss. RSC Adv..

[28] Sideridou I.D., Karabela M.M. (2009). Effect of the amount of 3-methacyloxypropyltrimethoxysilane coupling agent on physical properties of dental resin nanocomposites. Dent. Mater..

[29] Can-Karabulut D.C., Oz F.T., Karabulut B., Batmaz I., Ilk O. (2009). Adhesion to primary and permanent dentin and a simple model approach. Eur. J. Dent..

[30] Carvalho R.M., Manso A.P., Geraldeli S., Tay F.R., Pashley D.H. (2012). Durability of bonds and clinical success of adhesive restorations. Dent. Mater..

[31] Solhi L., Atai M., Nodehi A., Imani M. (2012). A novel dentin bonding system containing poly(methacrylic acid) grafted nanoclay: Synthesis, characterization and properties. Dent. Mater..

[32] Nobre C.M.G., Putz N., Hannig M. (2020). Adhesion of Hydroxyapatite Nanoparticles to Dental Materials under Oral Conditions. Scanning.

[33] Pepla E., Besharat L.K., Palaia G., Tenore G., Migliau G. (2014). Nano-hydroxyapatite and its applications in preventive, restorative and regenerative dentistry: A review of literature. Ann. Stomatol..

[34] Melo M.A., Cheng L., Zhang K., Weir M.D., Rodrigues L.K., Xu H.H. (2013). Novel dental adhesives containing nanoparticles of silver and amorphous calcium phosphate. Dent. Mater..

[35] Soares C.P.P., Baptista R.d.L., Cesar D.V. (2017). Solvothermal Reduction of Graphite Oxide Using Alcohols. Mater. Res..

[36] Mortazavi V., Fathi M., Ataei E., Khodaeian N., Askari N. (2012). Shear bond strengths and morphological evaluation of filled and unfilled adhesive interfaces to enamel and dentine. Int. J. Dent..

[37] Khan A.S., Farooq I., Alakrawi K.M., Khalid H., Saadi O.W., Hakeem A.S. (2020). Dentin Tubule Occlusion Potential of Novel Dentifrices Having Fluoride Containing Bioactive Glass and Zinc Oxide Nanoparticles. Med. Princ. Pract..

[38] Cantore S., Ballini A., Mori G., Dibello V., Marrelli M., Mirgaldi R., De Vito D., Tatullo M. (2016). Anti-plaque and antimicrobial efficiency of different oral rinses in a 3-day plaque accumulation model. J. Biol. Regul. Homeost. Agents.

[39] Spagnuolo G., Codispoti B., Marrelli M., Rengo C., Rengo S., Tatullo M. (2018). Commitment of Oral-Derived Stem Cells in Dental and Maxillofacial Applications. Dent. J..

[40] Ballini A., Cantore S., Scacco S., Coletti D., Tatullo M. (2018). Mesenchymal Stem Cells as Promoters, Enhancers, and Playmakers of the Translational Regenerative Medicine 2018. Stem Cells Int..

[41] Diniz I.M., Matos A.B., Marques M.M. (2015). Laser phototherapy enhances mesenchymal stem cells survival in response to the dental adhesives. Sci. World J..

[42] Anchieta R.B., Oliveira F.G., Sundfeld R.H., Rahal V., Machado L.S., Alexandre R.S., Sundefeld M.L., Rocha E.P. (2011). Analysis of hybrid layer thickness, resin tag length and their correlation with microtensile bond strength using a total etch adhesive to intact dentin. Acta Odontol. Ltinoam..

[43] Rangan S., Schulze H.G., Vardaki M.Z., Blades M.W., Piret J.M., Turner R.F.B. (2020). Applications of Raman spectroscopy in the development of cell therapies: State of the art and future perspectives. Analyst.

[44] Miyazaki M., Onose H., Iida N., Kazama H. (2003). Determination of residual double bonds in resin-dentin interface by Raman spectroscopy. Dent. Mater..

[45] Munther M., Shaygan M., Centeno A., Neumaier D., Zurutuza A., Momeni K., Davami K. (2019). Probing the mechanical properties of vertically-stacked ultrathin graphene/Al_2_O_3_ heterostructures. Nanotechnology.

[46] Nosenko V., Strutynska N., Vorona I., Zatovsky I., Dzhagan V., Lemishko S., Epple M., Prymak O., Baran N., Ishchenko S. (2015). Structure of Biocompatible Coatings Produced from Hydroxyapatite Nanoparticles by Detonation Spraying. Nano Res. Lett..

[47] Van Meerbeek B., Mohrbacher H., Celis J.P., Roos J.R., Braem M., Lambrechts P., Vanherle G. (1993). Chemical characterization of the resin-dentin interface by micro-Raman spectroscopy. J. Dent. Res..

[48] Sirisha K., Rambabu T., Ravishankar Y., Ravikumar P. (2014). Validity of bond strength tests: A critical review—Part II. J. Conserv. Dent..

[49] Wagner A., Belli R., Stotzel C., Hilpert A., Muller F.A., Lohbauer U. (2013). Biomimetically- and hydrothermally-grown HAp nanoparticles as reinforcing fillers for dental adhesives. J. Adhes. Dent..

[50] Khan A.A., Al-Khureif A.A., Saadaldin S.A., Mohamed B.A., Musaibah A.S.O., Divakar D.D., Eldwakhly E. (2019). Graphene oxide-based experimental silane primers enhance shear bond strength between resin composite and zirconia. Eur. J. Oral Sci..

[51] Helvatjoglu-Antoniades M., Koliniotou-Kubia E., Dionyssopoulos P. (2004). The effect of thermal cycling on the bovine dentine shear bond strength of current adhesive systems. J. Oral Rehab..

